# Supplementary material for: Suspension-Induced Stem Cell Transition: A Non-Transgenic Method to Generate Adult Stem Cells from Mouse and Human Somatic Cells
Source: Cells. 2023 Oct 23;12(20):2508. doi: 10.3390/cells12202508 (PMC10605402; doi:10.3390/cells12202508)
Supplement: Supplementary file 1 [file cells-12-02508-s001.zip › Captions for supplementary figures.pdf]

Suspension-Induced Stem Cell Transition: A Non-Transgenic Method to Generate Adult Stem Cells from Mouse and Human Somatic Cells

Authors: Behzad Yeganeh[1,2]*, Azadeh Yeganeh[3], Kyle Malone[1,4], Shawn T Beug[1,4,5], Robert P. Jankov[1,2,6]

Fig. S1

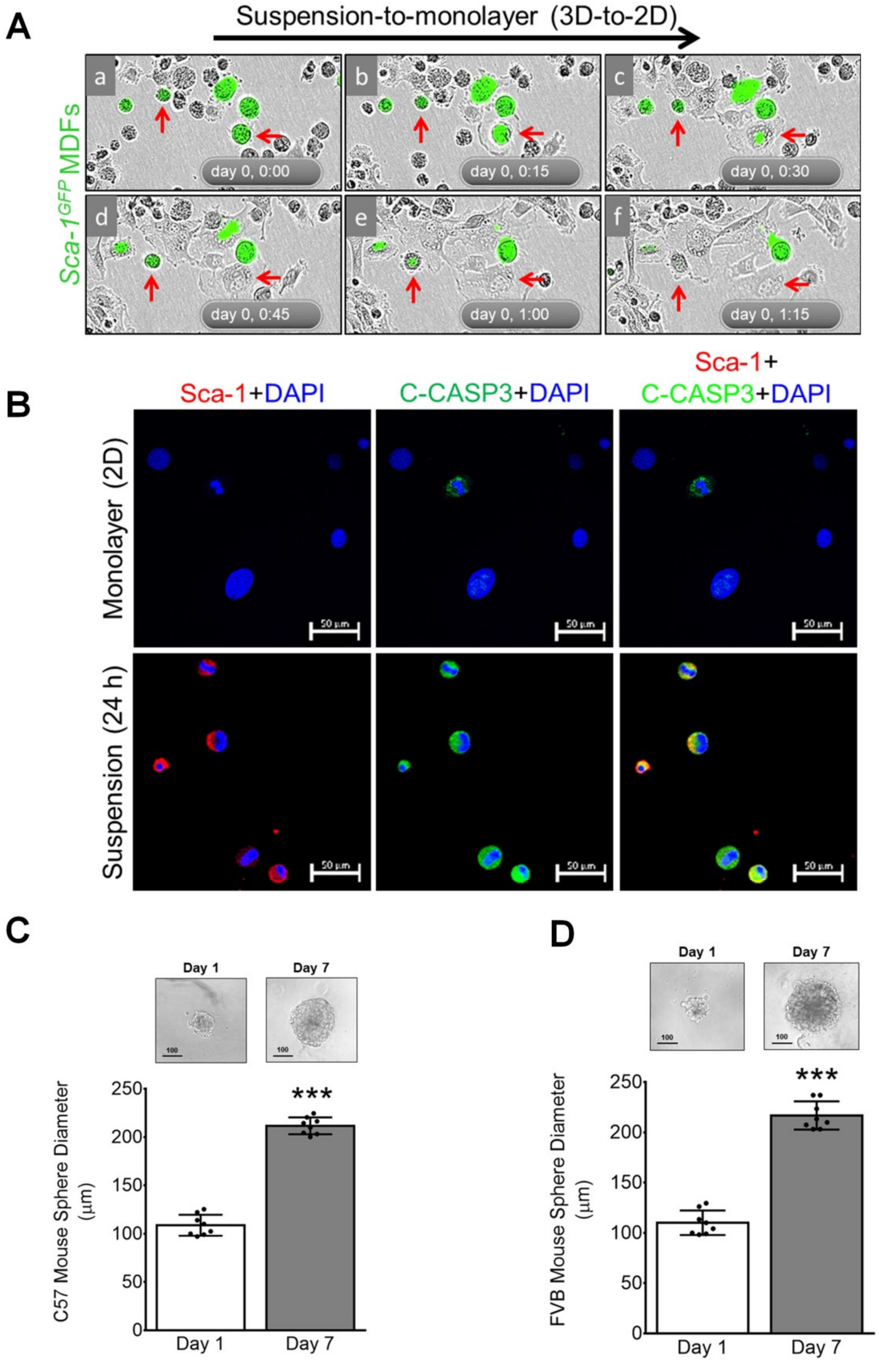
A
Suspension-to-monolayer (3D-to-2D)
Sca-1GFP MDFs
a
day 0, 0:00
b
day 0, 0:15
c
day 0, 0:30
d
day 0, 0:45
e
day 0, 1:00
f
day 0, 1:15
B
Sca-1+DAPI
C-CASP3+DAPI
Sca-1+
C-CASP3+DAPI
Monolayer (2D)
Suspension (24 h)
50 μm
C
Day 1
Day 7
C57 Mouse Sphere Diameter (μm)
250
200
150
100
50
0
***
Day 1
Day 7
D
Day 1
Day 7
FVB Mouse Sphere Diameter (μm)
250
200
150
100
50
0
***
Day 1
Day 7

Figure S1. Mouse TEFs grown in suspension culture and expressing stem cell markers undergo cell death. (A) Co-immunofluorescence staining of Sca-1 (red) with apoptosis marker cleaved Caspase 3 (C.CASP3) (green) in TEFs grown for 24 h in adherent (top) and suspension culture (bottom). Representative images of spheres of TEFs isolated from **(B)** C57 (top) and **(C)** FVB (bottom) wild-type mice and quantification of their spheroids size at day-7 compared to day one. (mean ± SEM, n = 8 spheroids). Scale bars are indicated in the images. ***p<0.001. Scale bars: 50 μm.

Fig. S2

A

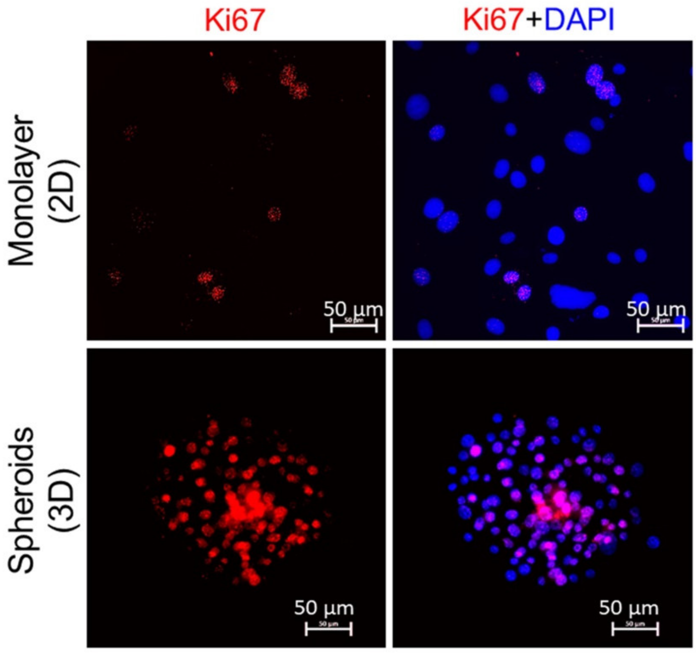
Ki67
Ki67+DAPI
Monolayer (2D)
Spheroids (3D)
50 µm
50 µm
50 µm
50 µm

B

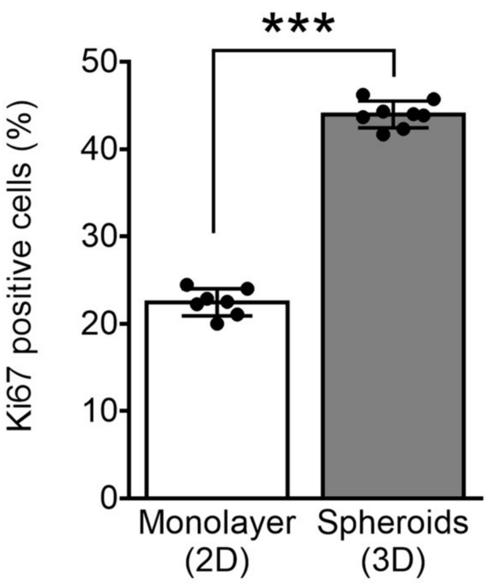
***
Ki67 positive cells (%)
50
40
30
20
10
0
Monolayer (2D)
Spheroids (3D)

Figure S2. Mouse TEF-derived spheroids are highly proliferative. (A) Representative IF staining for Ki67 expression in mouse fibroblasts grown in monolayer (2D) and spheroids grown in suspension culture (3D). Spheroids cultured for 7 days were stained for the presence of the proliferation marker Ki-67 (anti-Ki-67) and nuclei were stained with DAPI. **(B)** Quantitative analysis of Ki67-positive as a % of total cells. (mean ± SEM, n = 7-8 spheroids). Scale bars are indicated in the images. ***p<0.001.

Fig. S3

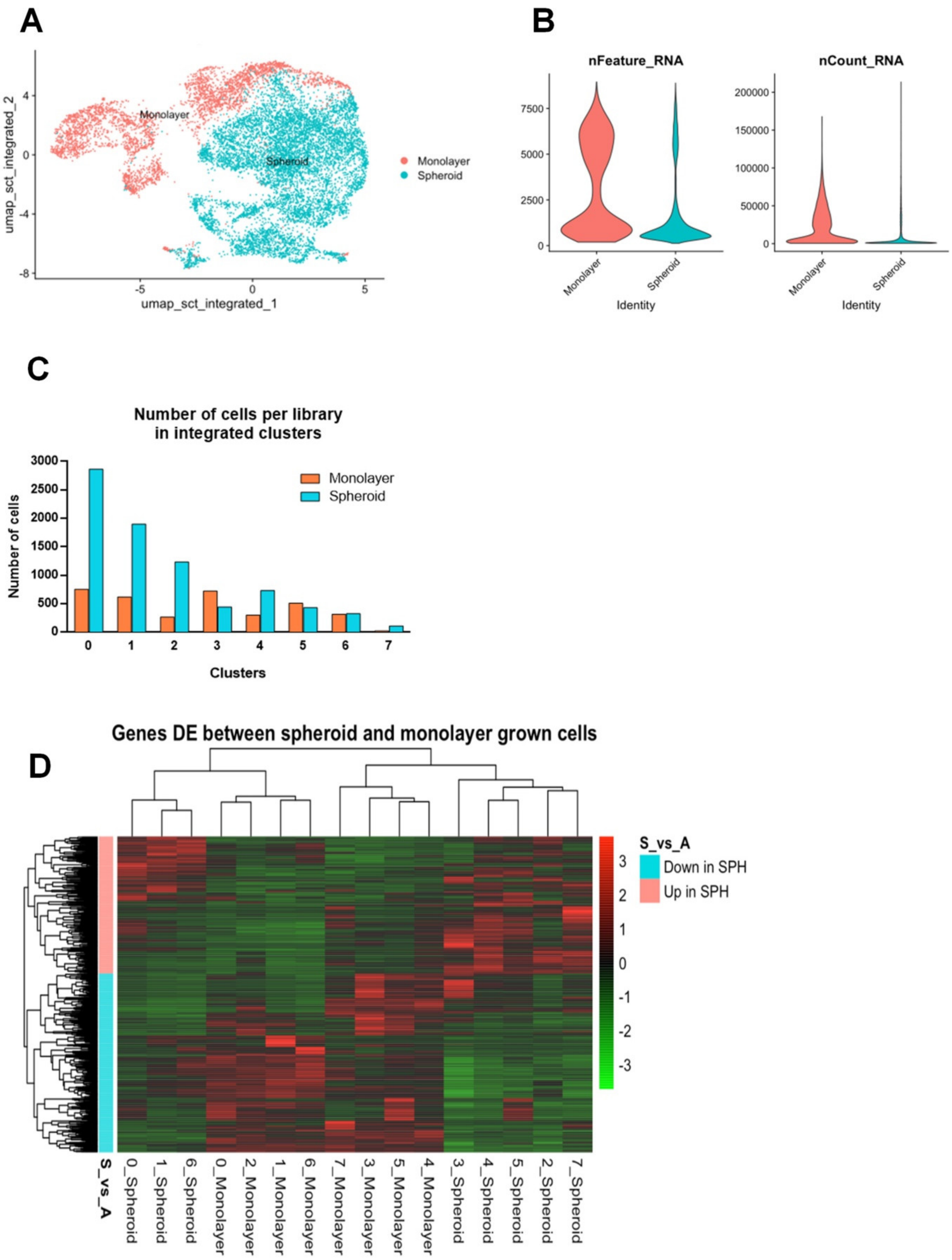
A
umap_sct_integrated_2
umap_sct_integrated_1
Monolayer
Spheroid
B
nFeature_RNA
nCount_RNA
Identity
C
Number of cells per library
in integrated clusters
Number of cells
Clusters
Monolayer
Spheroid
D
Genes DE between spheroid and monolayer grown cells
S_vs_A
Down in SPH
Up in SPH
0_Spheroid
1_Spheroid
6_Spheroid
0_Monolayer
2_Monolayer
1_Monolayer
6_Monolayer
7_Monolayer
3_Monolayer
5_Monolayer
4_Monolayer
3_Spheroid
4_Spheroid
5_Spheroid
2_Spheroid
7_Spheroid

Figure S3. Transcriptome analysis using RNA-seq in TEFs cultured as a monolayer and spheroids. (A) (A) UMAP projection of cells from the attached (monolayer) and suspension (spheroid) cells. UMAP embeddings calculated from a PCA reduction of the SCTransformed UMI counts, showing that the cells from the two libraries have generally non-overlapping UMAP embeddings. **(B)** Violin plots showing distributions of the number of detected genes (nFeature_RNA) and number of UMIs (nCount_RNA) per cell in the two libraries. Cells fall into two populations, with 'high' (> ~2,500) or 'low' (< ~2,500) numbers of detected genes. The absolute number of 'high' gene cells is similar in the two libraries, but the Spheroid library has a large excess of 'low' gene cells. (**B**) (**C**) Bar plot showing the number of cells from each library in each of the resolution 0.3 clusters identified on the integrated assay. (**D**) Heatmap illustrating differentially expressed genes (DEGs) of mouse TEFs cultured as a monolayer and spheroids in across 8 clusters, illustrating that each cluster exhibited unique gene expression.

Fig. S4

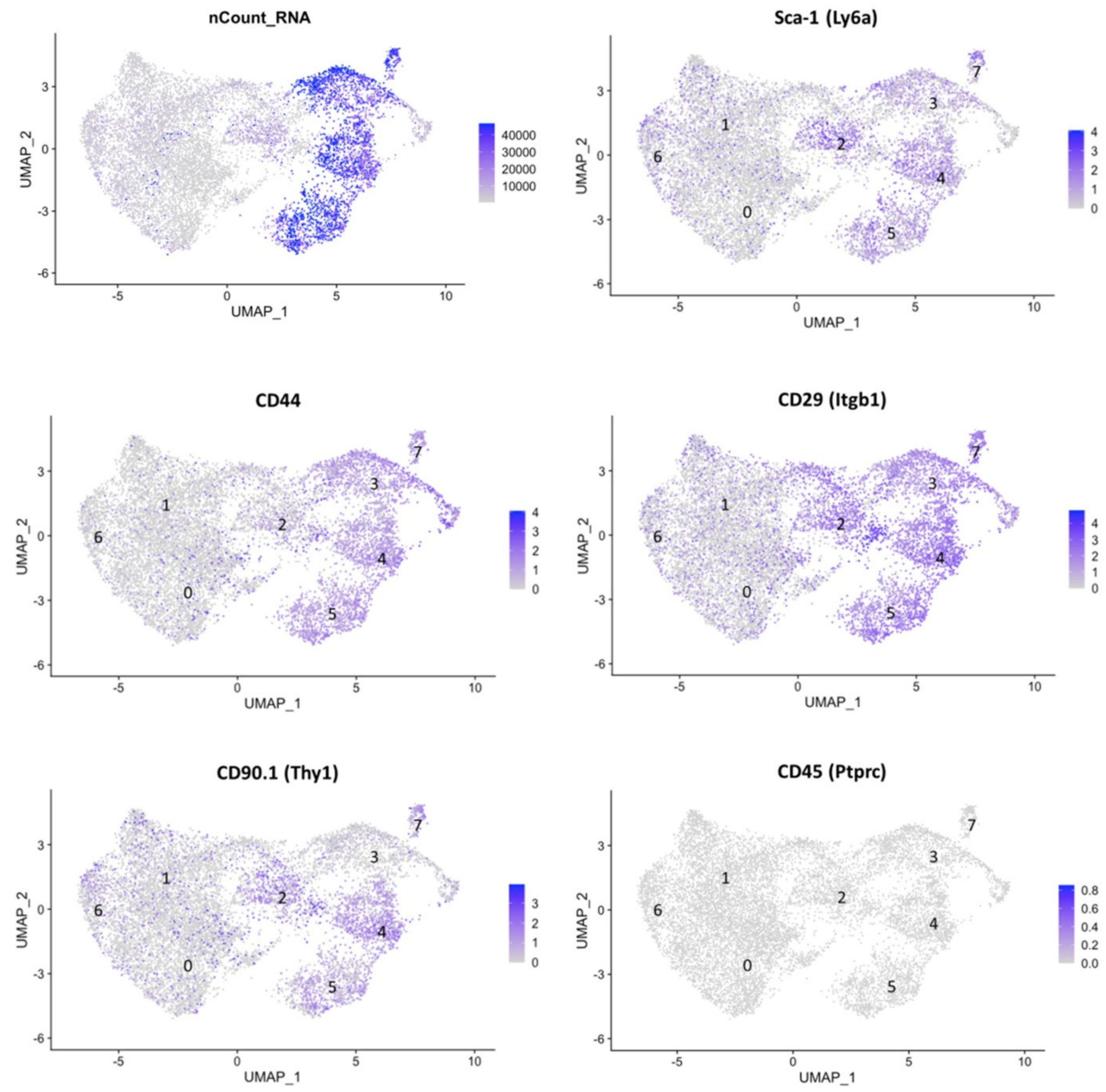
nCount_RNA
Sca-1 (Ly6a)
CD44
CD29 (Itgb1)
CD90.1 (Thy1)
CD45 (Ptprc)
UMAP_1
UMAP_2
40000
30000
20000
10000
0
1
2
3
4
5
6
7
0.8
0.6
0.4
0.2
0.0

Figure S4. Expression distribution for selected mouse MSCs-specific genes. (A) UMAP projections (integrated assay) showing the distribution of UMI counts, and the five MSC surface markers genes observed in IF staining in Figure 2D across the dataset (negative for *CD45 (Ptprc)* gene).

Fig. S5

A

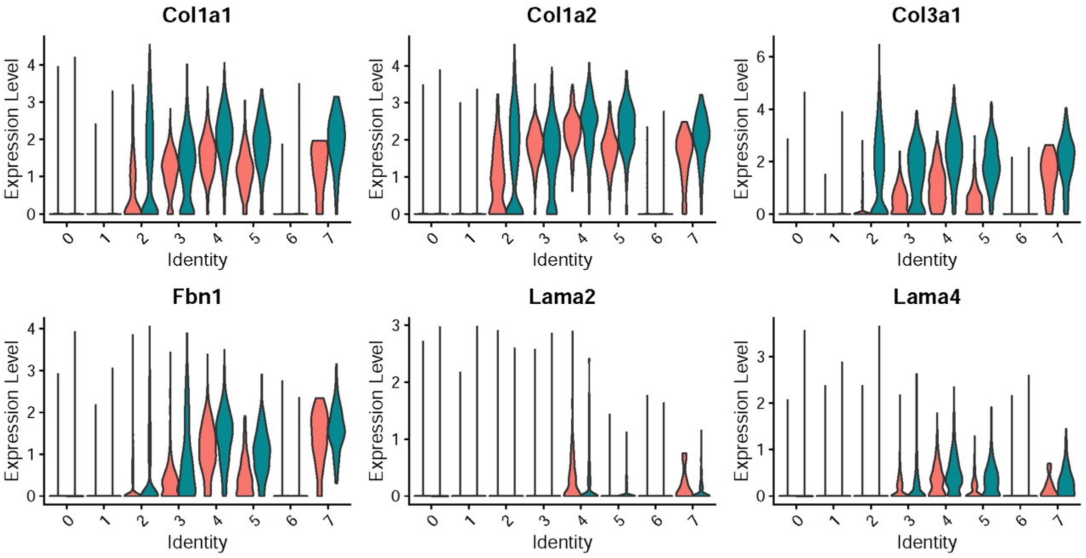
Col1a1
Col1a2
Col3a1
Fbn1
Lama2
Lama4
Expression Level
Identity

B

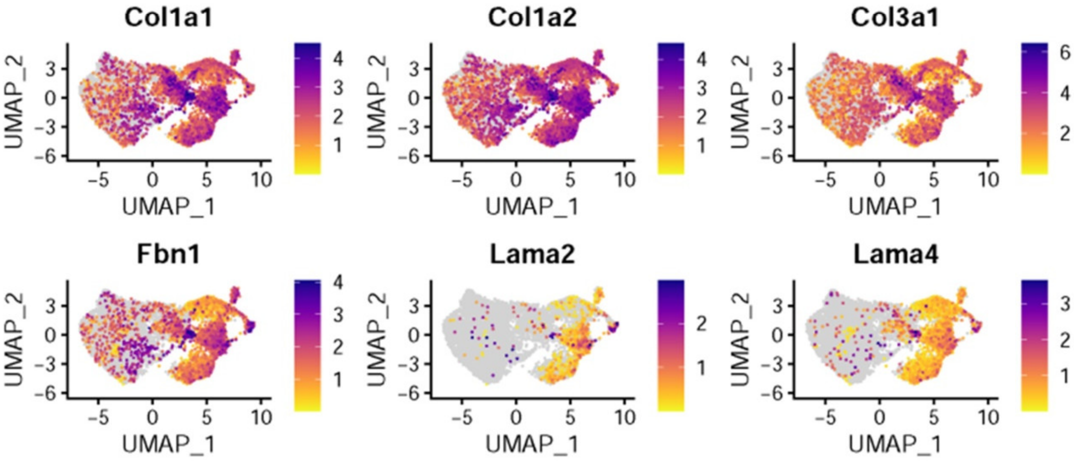
Col1a1
Col1a2
Col3a1
Fbn1
Lama2
Lama4
UMAP_2
UMAP_1

Figure S5. Expression distribution of selected ECM genes in monolayer and spheroid cells. (A) Violin plot illustrating expression of the six selected ECM genes in monolayer and spheroid cells across eight clusters. **(B)** Feature plots of expression distribution for six selected ECM genes with the lowest p-value in in the dataset. Expression levels for each cell are color-coded and overlaid onto the UMAP plot.

Fig. S6

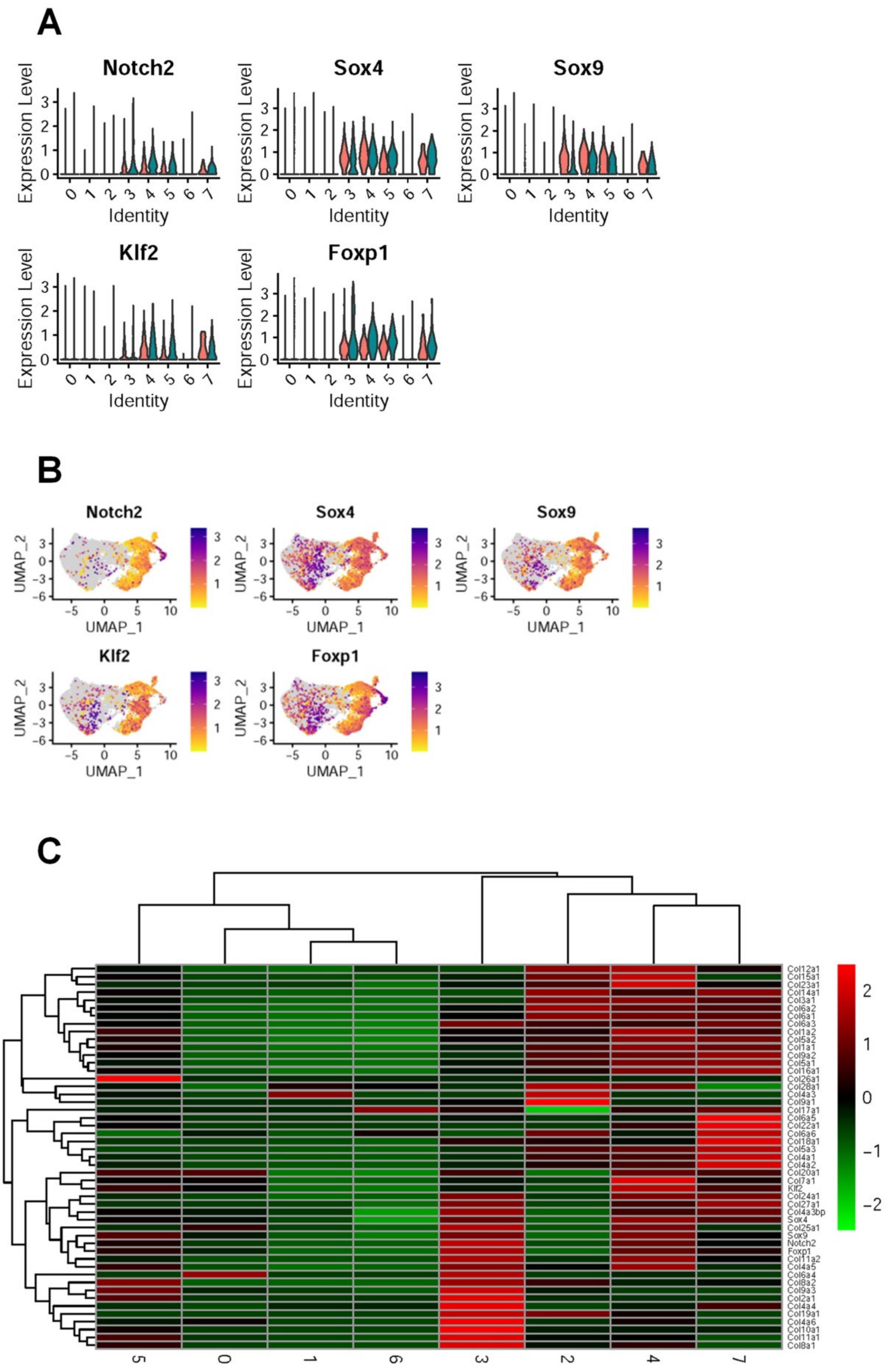
A
Notch2
Sox4
Sox9
Klf2
Foxp1
Expression Level
Identity
B
Notch2
Sox4
Sox9
Klf2
Foxp1
UMAP_2
UMAP_1
C
Col12a1
Col15a1
Col23a1
Col14a1
Col3a1
Col6a2
Col6a1
Col6a3
Col1a2
Col5a2
Col1a1
Col9a2
Col5a1
Col16a1
Col26a1
Col28a1
Col4a3
Col9a1
Col17a1
Col6a5
Col22a1
Col6a6
Col18a1
Col5a3
Col4a1
Col4a2
Col20a1
Col7a1
Klf2
Col24a1
Col27a1
Col4a3bp
Sox4
Col25a1
Sox9
Notch2
Foxp1
Col11a2
Col4a5
Col6a4
Col8a2
Col9a3
Col2a1
Col4a4
Col19a1
Col4a6
Col10a1
Col11a1
Col8a1
5
0
1
6
3
2
4
7

Figure S6. Expression distribution for self-renewal genes in monolayer and spheroid cells. (A) Violin plot showing expression of self-renewal genes *Notch2, Sox4, Sox9, Klf2*, and *Foxp1* across eight clusters. **(B)** Feature plots of expression distribution of self-renewal genes *Notch2, Sox4, Sox9, Klf2*, and *Foxp1*. Expression levels for each cell are color-coded and overlaid onto the UMAP plot. **(C)** Heatmap shows the average expression level of all collagen genes present in the dataset and selected self-renewal genes across clusters.

Fig. S7

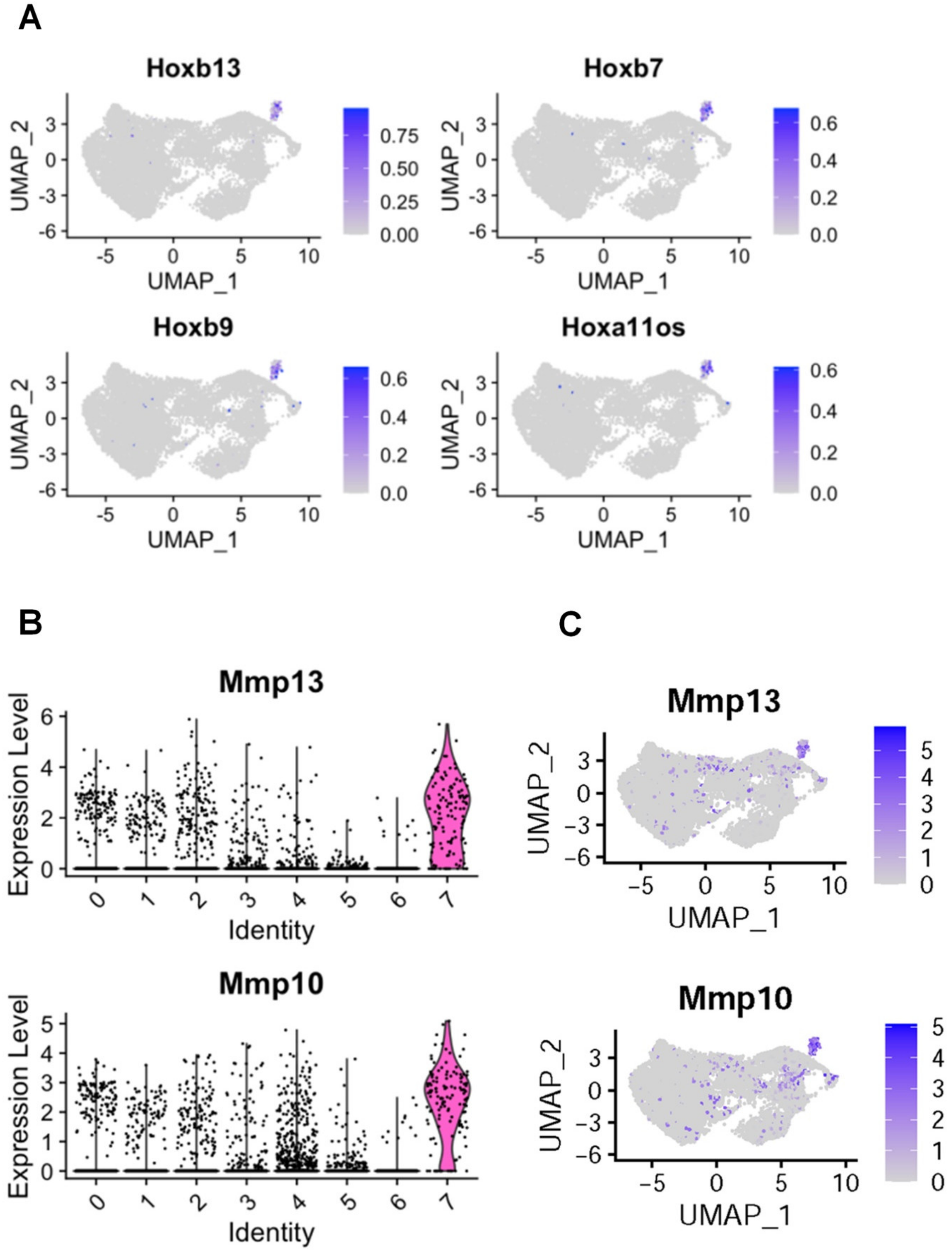
A
Hoxb13
Hoxb7
Hoxb9
Hoxa11os
UMAP_1
UMAP_2
B
Mmp13
Mmp10
Expression Level
Identity
C
Mmp13
Mmp10
UMAP_1
UMAP_2

Figure S7. Feature plots of expression distribution for *Hox* and *Mmp* genes. (A) UMAP visualization of expression distribution of selected *Hox* genes illustrating their higher expression in cluster 7. **(B)** Violin plot and (**C**) UMAP showing expression of *Mmp*13 and *Mmp*10 genes expressed more highly in cluster 7.

Fig. S8

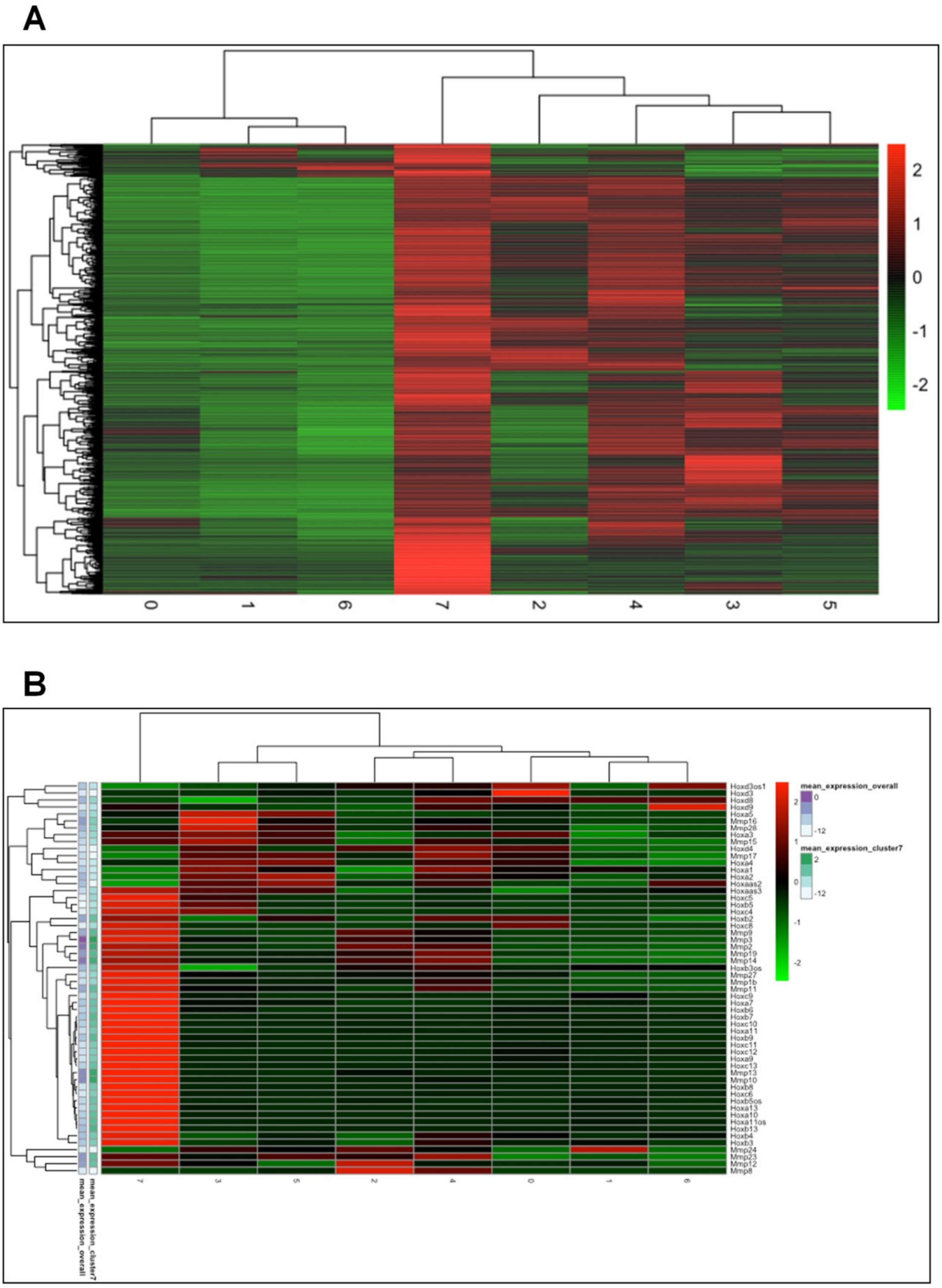
A
2
1
0
-1
-2
0
1
6
7
2
4
3
5
B
Hoxd3os1
Hoxd3
Hoxd8
Hoxd9
Hoxa5
Mmp16
Mmp28
Hoxa3
Mmp15
Hoxd4
Mmp17
Hoxa4
Hoxa1
Hoxa2
Hoxaas2
Hoxaas3
Hoxc5
Hoxb5
Hoxc4
Hoxb2
Hoxc8
Mmp9
Mmp3
Mmp2
Mmp19
Mmp14
Hoxb3os
Mmp27
Mmp1b
Mmp11
Hoxc9
Hoxa7
Hoxb6
Hoxb7
Hoxc10
Hoxa11
Hoxb9
Hoxc11
Hoxc12
Hoxa9
Hoxc13
Mmp13
Mmp10
Hoxb8
Hoxc6
Hoxb5os
Hoxa13
Hoxa10
Hoxa11os
Hoxb13
Hoxb4
Hoxb3
Mmp24
Mmp23
Mmp12
Mmp8
mean_expression_overall
0
-12
mean_expression_cluster7
2
-12
2
1
0
-1
-2
7
3
5
2
4
0
1
6
mean_expression_cluster7
mean_expression_overall

Figure S8. *Hox* and *Mmp* genes are highly expressed in cluster 7. (A) Heatmaps showing average normalized expression per cluster of genes expressed at a significantly higher level (adjusted p-value < 0.05) in cluster 7 than in all other cells in the dataset, in which clusters 4 and 7 look much more similar to each other; and **(B)** All *Hox* and *Mmp* genes with measured expression in the dataset. Normalized counts per cell were averaged for each cluster, and values were scaled and centered row-wise to plot Z-scores for each gene; rows and columns were hierarchically clustered using complete linkage clustering with a Euclidian distance metric. Sidebars in **(B)** show the log2 average normalized expression of each gene across the whole dataset and specifically in the cells in cluster 7.

Fig. S9

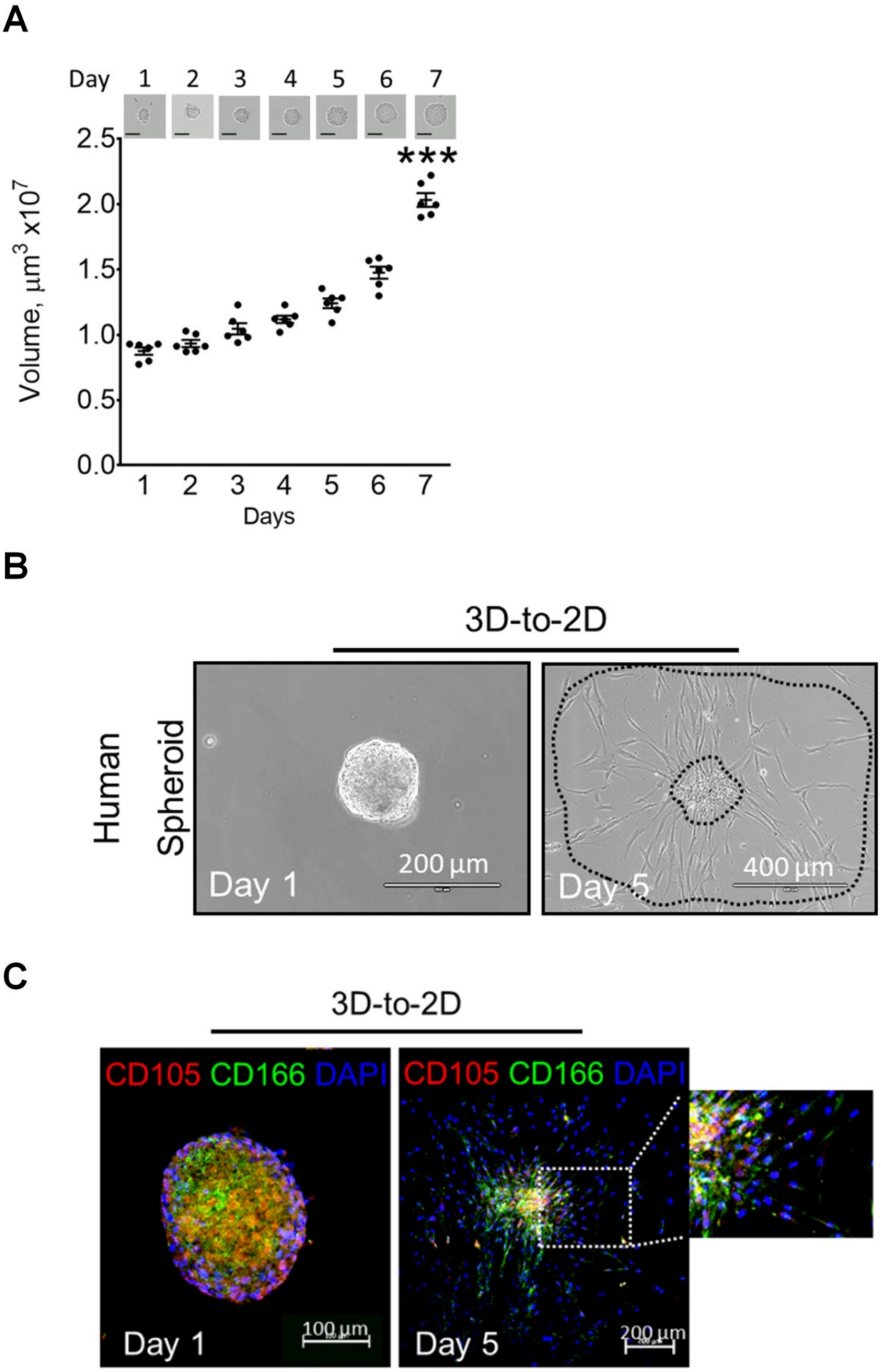
A
Day 1 2 3 4 5 6 7
Volume, µm3 x10^7
2.5
2.0
1.5
1.0
0.5
0.0
***
1 2 3 4 5 6 7
Days
B
3D-to-2D
Human Spheroid
Day 1
200 µm
Day 5
400 µm
C
3D-to-2D
CD105 CD166 DAPI
Day 1
100 µm
Day 5
200 µm

Figure S9. Human dermal fibroblast-derived spheroids are proliferative. (A) Quantification of spheroid volume using real-time cell imaging by IncuCyte® live-cell analysis system over a 7-day time course (n = 6). (B) A single sphere of human fibroblasts after seeding on a coverslip after 1 (left) and 5 days (right) in culture. (C) Co-immunofluorescence staining of CD105 (red) with another marker of human MSCs CD166 (green) in a single sphere after 1 (left panel) and 5 days in culture (right panel).
