# Supplementary material for: Suspension-Induced Stem Cell Transition: A Non-Transgenic Method to Generate Adult Stem Cells from Mouse and Human Somatic Cells
Source: Cells. 2023 Oct 23;12(20):2508. doi: 10.3390/cells12202508 (PMC10605402; doi:10.3390/cells12202508)
Supplement: Supplementary file 1 [file cells-12-02508-s001.zip › Supplementary Table 1.pdf]

TABLE S1: ECM genes

All of the six selected genes (*Col3a1, Col1a2, Col1a1, Fbn1, Lama4, Lama2*) are significantly higher in cluster 4 than in the remainder of the dataset. *Col1a2* is significantly higher in all of cluster 2, 3, 4, 5, and 7.

**Gene**	**Significant marker for clusters**
Col3a1	2, 4, 7
Col1a2	2, 3, 4, 5, 7
Col1a1	2, 4, 5, 7
Fbn1	3, 4, 7
Lama4	4
Lama2	4
