# Supplementary material for: Suspension-Induced Stem Cell Transition: A Non-Transgenic Method to Generate Adult Stem Cells from Mouse and Human Somatic Cells
Source: Cells. 2023 Oct 23;12(20):2508. doi: 10.3390/cells12202508 (PMC10605402; doi:10.3390/cells12202508)
Supplement: Supplementary file 1 [file cells-12-02508-s001.zip › Supplementary Table 2.pdf]

TABLE S2: Self-renewal genes

The self-renewal genes (*Foxp1, Sox9, Sox4, Fbn1, Notch2, Klf2*), are significantly higher in cluster 4 than in the remainder of the dataset.

Gene	Significant marker for clusters
Foxp1	3, 4, 5, 7
Sox9	3, 4, 5
Sox4	3, 4, 7
Notch2	3,4
Klf2	4
