# Supplementary material for: Suspension-Induced Stem Cell Transition: A Non-Transgenic Method to Generate Adult Stem Cells from Mouse and Human Somatic Cells
Source: Cells. 2023 Oct 23;12(20):2508. doi: 10.3390/cells12202508 (PMC10605402; doi:10.3390/cells12202508)
Supplement: Supplementary file 1 [file cells-12-02508-s001.zip › Supplementary Table 3 (GO terms of MSCs-related).pdf]

Supplementary Table 3: GO terms [GO Slim terms] for the genes displayed in Figure 4C and Figure S5 were downloaded from the Ensembl biomart repository using the R biomaRt package on 11th September, 2023.

GO Slim terms for the genes displayed in Figure 4C

mgi_symbol	GO slim
Cd44	cell adhesion | plasma membrane | signaling | molecular transducer activity | cell motility | carbohydrate derivative metabolic process | protein modification process | immune system process | programmed cell death | anatomical structure development | wound healing | organelle | Golgi apparatus | cytosol
Itgb1	anatomical structure development | programmed cell death | cell differentiation | signaling | plasma membrane | cytoplasmic vesicle | organelle | cell adhesion | vesicle-mediated transport | endosome | mitotic cell cycle | cell junction organization | nervous system process | cytoskeleton organization | cytoskeletal protein binding | protein modification process | wound healing | extracellular matrix | external encapsulating structure | cell motility | protein localization to plasma membrane | cell adhesion mediator activity | establishment or maintenance of cell polarity | reproductive process | immune system process | transmembrane transport | inflammatory response | membrane organization | intracellular protein transport | extracellular matrix organization
Ly6a	plasma membrane | molecular function regulator activity | signaling
Ptprc	catalytic activity | hydrolase activity | catalytic activity, acting on a protein | protein modification process | signaling | immune system process | anatomical structure development | cell differentiation | plasma membrane | defense response to other organism | transmembrane transport | metal ion homeostasis | vesicle-mediated transport | cytoskeletal protein binding | cell adhesion | programmed cell death | DNA recombination | cell motility | membrane organization
Thy1	cytosol | plasma membrane | cell adhesion | organelle | endoplasmic reticulum | molecular function regulator activity | immune system process | signaling | lipid binding | anatomical structure development | cytoskeleton organization | cell motility | protein modification process | transmembrane transport | metal ion homeostasis | cell differentiation | cell junction organization

The GO terms for Figure 4C: (GO terms of MSC-related)

mgi_symbol	GO
Cd44	membrane | cell adhesion | hyaluronic acid binding | cell projection | plasma membrane | cytokine-mediated signaling pathway | microvillus | cytokine receptor activity | cell migration | hyaluronan catabolic process | positive regulation of peptidyl-serine phosphorylation | positive regulation of heterotypic cell-cell adhesion | T cell activation | negative regulation of apoptotic process | negative regulation of cysteine-type endopeptidase activity involved in apoptotic process | negative regulation of DNA damage response, signal transduction by p53 class mediator | wound healing, spreading of cells | cellular response to fibroblast growth factor stimulus | positive regulation of peptidyl-tyrosine phosphorylation | cartilage development | positive regulation of ERK1 and ERK2 cascade | monocyte aggregation | positive regulation of monocyte aggregation | negative regulation of intrinsic apoptotic signaling pathway in response to DNA damage by p53 class mediator | regulation of lamellipodium morphogenesis | Golgi apparatus | cytosol | cell surface | apical plasma membrane | lamellipodium membrane | macrophage migration inhibitory factor receptor complex |
Itgb1	in utero embryonic development | negative regulation of apoptotic process | protein binding | positive regulation of angiogenesis | synapse | protein-containing complex binding | positive regulation of neuron projection development | positive regulation of

	neuron differentiation | axon extension | regulation of cell cycle | positive regulation of protein kinase B signaling | negative regulation of cell population proliferation | cytoplasm | tissue homeostasis | cell population proliferation | neuron differentiation | cell projection | membrane | metal ion binding | external side of plasma membrane | plasma membrane | cell surface | perinuclear region of cytoplasm | cytoplasmic vesicle | glutamatergic synapse | sarcolemma | lamellipodium | cell-cell junction | filopodium | focal adhesion | anchoring junction | cell adhesion | signaling receptor binding | protease binding | phagocytosis | endosome | recycling endosome | G1/S transition of mitotic cell cycle | integrin-mediated signaling pathway | kinase binding | muscle organ development | melanosome | calcium ion binding | protein heterodimerization activity | bicellular tight junction assembly | protein kinase binding | visual learning | sarcomere organization | ruffle membrane | actin binding | integrin binding | neuromuscular junction | protein domain specific binding | positive regulation of GTPase activity | dendrite morphogenesis | protein tyrosine kinase binding | positive regulation of peptidyl-tyrosine phosphorylation | positive regulation of apoptotic process | response to endogenous stimulus | collagen binding | positive regulation of cell-substrate adhesion | dendritic spine | cell junction | membrane raft | positive regulation of wound healing | cell-matrix adhesion | adherens junction | Schaffer collateral - CA1 synapse | alpha-actinin binding | neuron projection development | basement membrane | integrin complex | hemidesmosome | receptor complex | postsynaptic membrane | cell adhesion molecule binding | negative regulation of cell projection organization | cell adhesion mediated by integrin | positive regulation of neuroblast proliferation | neuroblast proliferation | regulation of G protein-coupled receptor signaling pathway | negative regulation of neuron differentiation | intercalated disc | cell projection organization | positive regulation of endocytosis | modulation of chemical synaptic transmission | ruffle | cardiac muscle cell differentiation | magnesium ion binding | cell migration involved in sprouting angiogenesis | glial cell projection | negative regulation of Rho protein signal transduction | laminin binding | integrin alpha9-beta1 complex | positive regulation of protein localization to plasma membrane | positive regulation of cell migration | acrosomal vesicle | synaptic membrane | collagen binding involved in cell-matrix adhesion | integrin alpha10-beta1 complex | establishment of mitotic spindle orientation | cell-substrate adhesion | fibronectin binding | positive regulation of signaling receptor activity | myelin sheath abaxonal region | C-X3-C chemokine binding | positive regulation of fibroblast migration | heterotypic cell-cell adhesion | maintenance of postsynaptic specialization structure | regulation of postsynaptic neurotransmitter receptor diffusion trapping | glycinergic synapse | lamellipodium assembly | negative regulation of anoikis | leukocyte cell-cell adhesion | regulation of synapse pruning | cerebellar climbing fiber to Purkinje cell synapse | germ cell migration | leukocyte tethering or rolling | positive regulation of glutamate uptake involved in transmission of nerve impulse | regulation of inward rectifier potassium channel activity | regulation of spontaneous synaptic transmission | reactive gliosis | calcium-independent cell-matrix adhesion | myoblast fusion | regulation of collagen catabolic process | formation of radial glial scaffolds | CD40 signaling pathway | receptor internalization | protein transport within lipid bilayer | cell-cell adhesion mediated by integrin | myoblast differentiation | mesodermal cell differentiation | myoblast fate specification | cardiac muscle cell myoblast differentiation | cardiac cell fate specification | cellular response to low-density lipoprotein particle stimulus | basement membrane organization | integrin alpha1-beta1 complex | integrin alpha2-beta1 complex | integrin alpha3-beta1 complex | integrin alpha5-beta1 complex | integrin alpha7-beta1 complex | integrin alpha11-beta1 complex |
Ly6a	response to bacterium | membrane | plasma membrane | external side of plasma membrane | protein binding | acetylcholine receptor inhibitor activity | acetylcholine receptor binding | acetylcholine receptor signaling pathway | synapse |

Ptprc	| protein binding | phosphatase activity | protein tyrosine phosphatase activity | protein dephosphorylation | dephosphorylation | T cell receptor signaling pathway | regulation of gene expression | positive regulation of stem cell proliferation | stem cell development | bone marrow development | membrane | external side of plasma membrane | cell periphery | plasma membrane | hydrolase activity | heparin binding | cell surface | membrane raft | defense response to virus | phosphoprotein phosphatase activity | focal adhesion | regulation of cell cycle | positive regulation of B cell proliferation | B cell proliferation | positive regulation of MAPK cascade | B cell differentiation | release of sequestered calcium ion into cytosol | protein kinase binding | hematopoietic progenitor cell differentiation | regulation of phagocytosis | spectrin binding | ankyrin binding | MAPK cascade | gamma-delta T cell differentiation | positive thymic T cell selection | positive regulation of T cell differentiation | positive regulation of gamma-delta T cell differentiation | protein autophosphorylation | heparan sulfate proteoglycan binding | positive regulation of MAP kinase activity | negative regulation of protein autophosphorylation | regulation of receptor signaling pathway via JAK-STAT | T cell proliferation | negative regulation of protein kinase activity | response to gamma radiation | T cell differentiation | positive regulation of immunoglobulin production | peptidyl-tyrosine phosphorylation | positive regulation of extrinsic apoptotic signaling pathway | negative regulation of T cell mediated cytotoxicity | signaling receptor binding | positive regulation of tumor necrosis factor production | heterotypic cell-cell adhesion | regulation of protein tyrosine kinase activity | extrinsic apoptotic signaling pathway | positive regulation of T cell proliferation | alpha-beta T cell proliferation | positive regulation of alpha-beta T cell proliferation | leukocyte cell-cell adhesion | B cell receptor signaling pathway | positive regulation of isotype switching to IgG isotypes | positive regulation of T cell mediated cytotoxicity | negative thymic T cell selection | positive regulation of peptidyl-tyrosine phosphorylation | calcium-mediated signaling using intracellular calcium source | negative regulation of peptidyl-tyrosine phosphorylation | natural killer cell differentiation | negative regulation of cytokine-mediated signaling pathway | positive regulation of T cell mediated immunity | regulation of humoral immune response mediated by circulating immunoglobulin | positive regulation of humoral immune response mediated by circulating immunoglobulin | negative regulation of cell adhesion involved in substrate-bound cell migration | cellular response to extracellular stimulus | regulation of interleukin-8 production | negative regulation of interleukin-2 production | positive regulation of interleukin-2 production | cell cycle phase transition | plasma membrane raft distribution | positive regulation of antigen receptor-mediated signaling pathway | negative regulation of protein tyrosine kinase activity | negative regulation of ERK1 and ERK2 cascade | positive regulation of ERK1 and ERK2 cascade | positive regulation of protein tyrosine phosphatase activity | positive regulation of Fc-gamma receptor signaling pathway involved in phagocytosis | positive regulation of hematopoietic stem cell migration | regulation of extrinsic apoptotic signaling pathway | cytoplasmic side of plasma membrane | bleb | membrane microdomain
Thy1	cytosol | membrane | external side of plasma membrane | plasma membrane | cell surface | apical plasma membrane | growth cone | cell-cell adhesion | endoplasmic reticulum | GTPase activator activity | positive regulation of T cell activation | integrin-mediated signaling pathway | GPI anchor binding | dendrite | positive regulation of GTPase activity | angiogenesis | T cell receptor signaling pathway | protein kinase binding | cytoskeleton organization | integrin binding | cell-cell signaling | membrane raft | myelin sheath | enzyme binding | negative regulation of cell migration | receptor clustering | neuronal cell body membrane | protein autophosphorylation | dendrite membrane | positive regulation of heterotypic cell-cell adhesion | positive regulation of release of sequestered calcium ion into cytosol | negative regulation of protein kinase activity | negative regulation of axonogenesis | regulation of cell-matrix adhesion |

	negative regulation of protein tyrosine kinase activity | positive regulation of focal adhesion assembly | focal adhesion assembly | positive regulation of cellular extravasation | retinal cone cell development | negative regulation of T cell receptor signaling pathway | negative regulation of neuron projection regeneration | regulation of Rho-dependent protein serine/threonine kinase activity | axolemma |
