# Supplementary material for: Suspension-Induced Stem Cell Transition: A Non-Transgenic Method to Generate Adult Stem Cells from Mouse and Human Somatic Cells
Source: Cells. 2023 Oct 23;12(20):2508. doi: 10.3390/cells12202508 (PMC10605402; doi:10.3390/cells12202508)
Supplement: Supplementary file 1 [file cells-12-02508-s001.zip › Supplementary Table 4 (GO terms of ECM-related).pdf]

Supplementary Table 4: GO terms [GO Slim terms] for the genes displayed in Figure 4C and Figure S5 were downloaded from the Ensembl biomart repository using the R biomaRt package on 11th September, 2023.

The GO terms for Figure S5: GO terms of ECM-related

**mgi_symbol**	**GO**
Col1a1	structural molecule activity | nervous system process | anatomical structure development | extracellular region | extracellular space | organelle | Golgi apparatus | endoplasmic reticulum | **extracellular matrix** | external encapsulating structure | DNA-templated transcription | regulation of DNA-templated transcription | cytoplasmic vesicle | extracellular matrix organization | signaling | cell adhesion | cell motility | cell differentiation | wound healing
Col1a2	structural molecule activity | signaling | circulatory system process | anatomical structure development | extracellular region | extracellular space | molecular adaptor activity | extracellular matrix organization | **extracellular matrix** | external encapsulating structure | protein-containing complex assembly
Col3a1	structural molecule activity | anatomical structure development | signaling | extracellular region | extracellular space | **extracellular matrix** | external encapsulating structure | extracellular matrix organization | cell differentiation | cell motility | cell adhesion | protein modification process | wound healing | immune system process
Fbn1	carbohydrate metabolic process | anatomical structure development | extracellular region | extracellular space | **extracellular matrix** | external encapsulating structure | immune system process | cell differentiation | molecular function regulator activity | receptor ligand activity | structural molecule activity | cell adhesion | signaling | protein modification process
Lama2	cell adhesion | cell motility | anatomical structure development | extracellular region | plasma membrane | **extracellular matrix** | external encapsulating structure | structural molecule activity | cell differentiation | signaling | extracellular matrix organization
Lama4	cell adhesion | cell motility | anatomical structure development | cell differentiation | extracellular region | **extracellular matrix** | external encapsulating structure | structural molecule activity

mgi_symbol	GO
Col1a1	protein binding | extracellular matrix structural constituent | sensory perception of sound | cellular response to amino acid stimulus | cytoplasm | skeletal system development | skin development | metal ion binding | extracellular space | Golgi apparatus | extracellular region | endoplasmic reticulum | collagen trimer | extracellular matrix | protease binding | positive regulation of DNA-templated transcription | identical protein binding | anatomical structure development | skeletal system morphogenesis | face morphogenesis | secretory granule | extracellular matrix organization | collagen fibril organization | blood vessel development | skin morphogenesis | extracellular matrix structural constituent conferring tensile strength | collagen-containing extracellular matrix | embryonic skeletal system development | positive regulation of canonical Wnt signaling pathway | protein transport | collagen-activated tyrosine kinase receptor signaling pathway | negative regulation of cell-substrate adhesion | positive regulation of cell migration | intramembranous ossification | visual perception | collagen biosynthetic process | protein localization to nucleus | tooth mineralization | positive regulation of epithelial to mesenchymal transition | wound healing | ossification | osteoblast differentiation | platelet-derived growth factor binding | endochondral ossification | response to nutrient | response to xenobiotic stimulus | response to mechanical stimulus |

	response to organic cyclic compound | response to nutrient levels | response to estradiol | response to insulin | response to hydrogen peroxide | response to peptide hormone | cellular response to fibroblast growth factor stimulus | response to steroid hormone | response to cAMP | response to hyperoxia | bone trabecula formation | cartilage development involved in endochondral bone morphogenesis | cellular response to mechanical stimulus | cellular response to retinoic acid | cellular response to vitamin E | cellular response to tumor necrosis factor | cellular response to epidermal growth factor stimulus | cellular response to transforming growth factor beta stimulus | response to fluoride | cellular response to fluoride | collagen type I trimer |
Col1a2	| extracellular matrix structural constituent | cellular response to amino acid stimulus | protein binding | transforming growth factor beta receptor signaling pathway | regulation of blood pressure | skeletal system development | metal ion binding | extracellular space | extracellular region | collagen trimer | protease binding | identical protein binding | protein-macromolecule adaptor activity | extracellular matrix organization | extracellular matrix | Rho protein signal transduction | bone mineralization | collagen fibril organization | collagen metabolic process | blood vessel development | SMAD binding | skin morphogenesis | extracellular matrix structural constituent conferring tensile strength | collagen-containing extracellular matrix | platelet-derived growth factor binding | extracellular matrix assembly | collagen type I trimer | protein heterotrimerization
Col3a1	| protein binding | extracellular matrix structural constituent | in utero embryonic development | gene expression | lung development | cellular response to amino acid stimulus | multicellular organism growth | transforming growth factor beta receptor signaling pathway | heart development | tissue homeostasis | cartilage development | skin development | metal ion binding | extracellular space | extracellular region | collagen trimer | extracellular matrix | protease binding | anatomical structure development | extracellular matrix organization | integrin binding | neuron migration | aorta development | cell-matrix adhesion | collagen fibril organization | fibroblast proliferation | elastic fiber assembly | blood vessel development | SMAD binding | chondrocyte differentiation | extracellular matrix structural constituent conferring tensile strength | collagen-containing extracellular matrix | digestive tract development | cerebral cortex development | peptide cross-linking | response to cytokine | integrin-mediated signaling pathway | positive regulation of Rho protein signal transduction | response to radiation | wound healing | response to angiotensin | layer formation in cerebral cortex | supramolecular fiber organization | platelet-derived growth factor binding | basement membrane organization | negative regulation of immune response | skeletal system development | response to mechanical stimulus | transforming growth factor beta1 production | limb joint morphogenesis | endochondral bone morphogenesis | aorta smooth muscle tissue morphogenesis | negative regulation of neuron migration | collagen type III trimer
Fbn1	calcium ion binding | protein binding | glucose metabolic process | protein-containing complex binding | heart development | skeletal system development | extracellular space | heparin binding | extracellular region | extracellular matrix | negative regulation of osteoclast differentiation | identical protein binding | hormone activity | glucose homeostasis | kidney development | integrin binding | extracellular matrix structural constituent | basement membrane | cell adhesion mediated by integrin | collagen-containing extracellular matrix | metanephros development | protein kinase A signaling | microfibril | sequestering of BMP in extracellular matrix | negative regulation of osteoclast development | activation of protein kinase A activity | sequestering of TGFbeta in extracellular matrix | | camera-type eye development | embryonic eye morphogenesis | post-embryonic eye morphogenesis | cellular response to transforming growth factor beta stimulus | cellular response to insulin-like growth factor stimulus
Lama2	| cell adhesion | signaling receptor binding | regulation of cell adhesion | regulation of

	cell migration | regulation of embryonic development | protein binding | extracellular region | sarcolemma | basement membrane | extracellular matrix structural constituent | dendritic spine | collagen-containing extracellular matrix | axon guidance | synaptic cleft | neuromuscular junction | positive regulation of synaptic transmission, cholinergic | positive regulation of cell adhesion | positive regulation of muscle cell differentiation | regulation of basement membrane organization | positive regulation of integrin-mediated signaling pathway | protein complex involved in cell-matrix adhesion | Schwann cell differentiation
Lama4	| cell adhesion | signaling receptor binding | regulation of cell adhesion | regulation of cell migration | regulation of embryonic development | brown fat cell differentiation | extracellular region | basement membrane | negative regulation of cold-induced thermogenesis | blood vessel development | collagen-containing extracellular matrix | extracellular matrix structural constituent | synaptic cleft | neuromuscular junction
