# Supplementary material for: Suspension-Induced Stem Cell Transition: A Non-Transgenic Method to Generate Adult Stem Cells from Mouse and Human Somatic Cells
Source: Cells. 2023 Oct 23;12(20):2508. doi: 10.3390/cells12202508 (PMC10605402; doi:10.3390/cells12202508)
Supplement: Supplementary file 1 [file cells-12-02508-s001.zip › Supplementary Table S5.pdf]

TABLE S5: KEY RESOURCES

REAGENT	DILUTION	SOURCE	IDENTIFIER
**Antibodies**			
Cleaved Caspase 3	1:100	Cell Signaling	9664
CD117 (c-Kit)	1:100	Novus Biologicals	AF1356
Sca-1/Ly6	1:150	R&D systems	MAB1226
CD44 (1M7.8.1)	1:100	Novus Biologicals	NBP2-22436
CD90.1	1:100	Novus Biologicals	NBP2-00312
CD29/ Integrin beta 1	1:50	Novus Biologicals	NBP2-36561
CD45R (RA3-6B2)	1:100	Novus Biologicals	NB100-77420
Ki-67 (D3B5)	1:50	NEB	12202T
CD133	1:200	Proteintech	66666-1-Ig
Alpha-Fetoprotein/AFP	1:100	Novus Biologicals	NBP1-76275
Albumin	1:100	Novus Biologicals	AF3329
STRO-1	1:50	Novus Biologicals	NBP1-48356
CD105/ Endoglin	1:50	Novus Biologicals	NBP2-22122
VCAM-1/CD106	1:50	R&D systems	MAB8091
CD146/MCAM	1:100	Novus Biologicals	AF932
CD19	1:100	Novus Biologicals	NBP2-25196
Human ALCAM/CD166	1:100	R&D systems	MAB6563
EpCAM (D4K8R)	1:50	NEB	36746T
Cytokeratin 7	1:100	Novus Biologicals	NBP2-44814
Cytokeratin 19	1:100	Novus Biologicals	NB100-687
Human p63/TP73L	1:100	R&D systems	AF1916
CD71/Human TfR (Transferrin R)	1:100	R&D systems	AF2474
Cytokeratin 1	1:100	Novus Biologicals	NB100-2756
Keratin 5 (E2T4B) XP	1:100	NEB	71536
Keratin 19 (BA17)	1:100	NEB	4558
Cleaved Caspase-3 (Asp175)	1:150	NEB	9661S
Cytokeratin 14	1:50	Proteintech	10143-1-AP
CD73	1:100	Novus Biological	NBP2-48480SS
CD45	1:100	Life Technologies	14-0459-82
GFP tag	1:1000	Proteintech	66002-1-Ig
Cy5 AffiniPure Donkey Anti-Mouse IgG (H+L)	1:200	Jackson ImmunoResearch	715-175-151
Cy5 AffiniPure Donkey Anti-Rabbit IgG (H+L)	1:200	Jackson ImmunoResearch	711-175-152
Alexa Fluor Donkey anti goat 488	1:200	Invitrogen	A32814
Alexa Fluor Goat anti mouse 488	1:200	Invitrogen	A32723
Alexa Fluor Goat anti rabbit 488	1:200	Invitrogen	A-11008
Alexa Fluor Goat anti mouse 546	1:200	Invitrogen	A-11030
Alexa Fluor Donkey anti goat 546	1:200	Invitrogen	A-11056
Alexa Fluor Donkey anti rat 488	1:200	Invitrogen	A48269

**Recombinant Growth Factors**		
Recombinant Murine bFGF	PeproTech	450-33
Recombinant Murine EGF	Stemcell technologies	78016.1
Recombinant Murine IGF-I	Stemcell technologies	78078.1
Human Recombinant bFGF	Stemcell technologies	78003
Human Recombinant EGF	Stemcell technologies	78006.1
Human Recombinant IGF-I	Stemcell technologies	78022.1
Recombinant Human HGF	PeproTech	100-39H
Mouse Collagen IV	R&D systems	3410-010-02
Human Collagen IV	Sigma-Aldrich	C7521
**Plates**		
Costar 6-well Clear Flat Bottom ULA Plate	Corning	3471
Corning 96-well Clear Round Bottom ULA Plate	Corning	7007
96 well tissue culture Flat Bottom Plates	Fisher Scientific	0720089
Nunc Lab-Tek Chamber Slide System- 2 wells	Fisher Scientific	177380
Nunc Lab-Tek Chamber Slide System- 8 wells	Fisher Scientific	177402
Normal Human Liver slides	TissueArray.Com	HuFPT074
**Chemicals**		
Dexamethasone	Cayman Chemical	11015
3-Isobutyl-1-methylxanthine	Cayman Chemical	13347
Y-27632 (hydrochloride)	Cayman Chemical	10005583
1-Oleoyl Lysophosphatidic Acid	Cayman Chemical	10010093-1
Sphingosine-1-phosphate (S1P)	Cayman Chemical	62570-1
A 83-01	Cayman Chemical	9001799-5
CHIR99021	Cayman Chemical	13122-1
Ascorbic Acid	Sigma-Aldrich	A8960
BGP (β-Glycererol Phosphate)	Sigma-Aldrich	G9891
Indomethacin	Sigma-Aldrich	I8280
3-Isobutyl-1-methylxanthine (IBMX)	Cayman Chemical	13347
**Media/supplements**		
William's E Medium	Fisher Scientific	A1217601
StemXVivo Osteogenic/Adipogenic Base Media	R&D systems	CCM007
Gibco DMEM/F-12	Fisher Scientific	11-330-032
ITS Liquid Media Supplement (100×)	Sigma-Aldrich	I3146-5ML
Primary Hepatocyte Maintenance Supplements	Fisher Scientific	CM4000
EpiLife Medium, with 60 µM calcium	Fisher Scientific	MEPI500CA
Human Keratinocyte Growth Supplement	Fisher Scientific	S-001-5
Penicillin-Streptomycin	Fisher Scientific	15140122
L-Glutamine	Fisher Scientific	25030081
Normal Human Liver slides	TissueArray.Com	HuFPT074

**Experimental Models: Strains/Cells**		
C57BL/6J mice	The Jackson Laboratory	000664
FVB/NJ mice	The Jackson Laboratory	001800
Ly6a-GFP mice	The Jackson Laboratory	012643
Primary Human Fibroblast, Skin (Arm), 11 YR	Coriell Institute for Medical Research	GM00323
Primary Human Fibroblast, Skin, 9 YR	Coriell Institute for Medical Research	GM00038
Primary Human Dermal Fibroblast (49 YR)	Zenbio	DDFM063011A
Primary Human Hepatocytes (25 YR)	Zenbio	Catalog# HPF; lot# ZBH1989-P
Primary Human Hepatocytes (31 YR)	Sigma-Aldrich	MTOXH1001; lot# LHuf15102
Human Keratinocytes from Skin, Foreskin	Coriell Institute for Medical Research	GM22267
Human Epidermal Keratinocytes, adult (HEKa)	Fisher Scientific	C0055C
