# Supplementary figures and images for: Suspension-Induced Stem Cell Transition: A Non-Transgenic Method to Generate Adult Stem Cells from Mouse and Human Somatic Cells
Source: Cells. 2023 Oct 23;12(20):2508. doi: 10.3390/cells12202508 (PMC10605402; doi:10.3390/cells12202508)

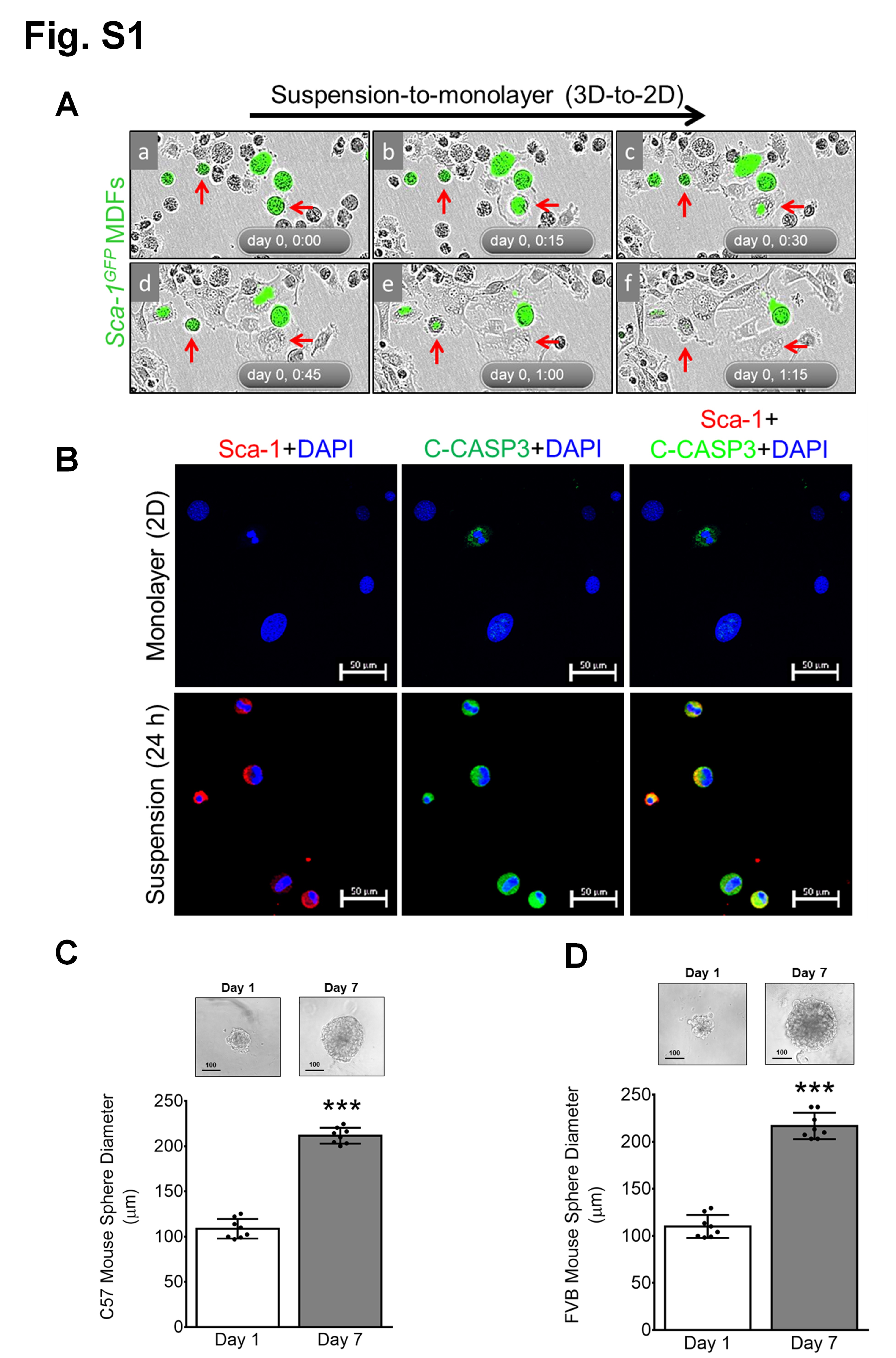

Supplement: Supplementary file 1 [file cells-12-02508-s001.zip › Fig. S1.jpg]

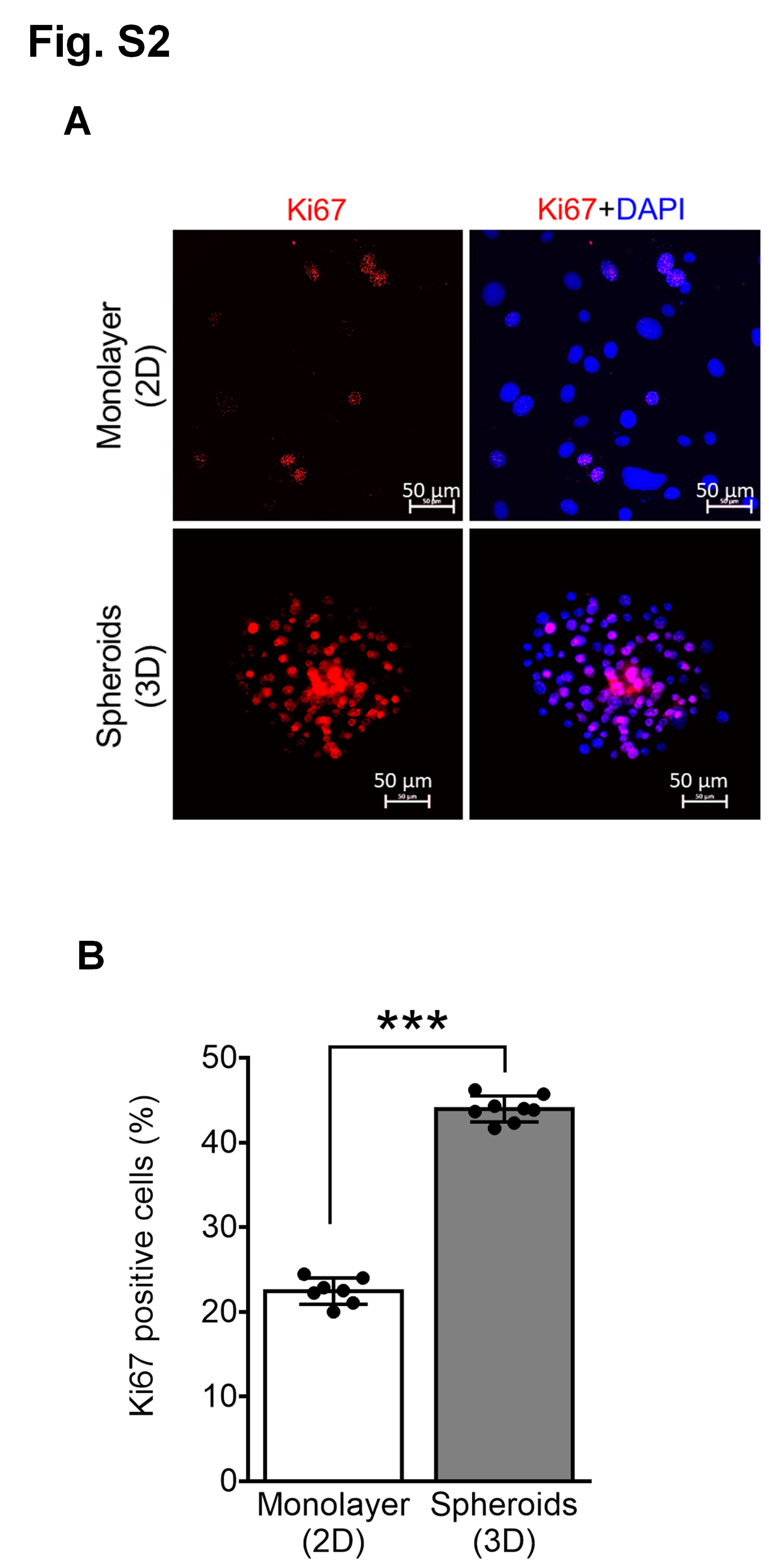

Supplement: Supplementary file 1 [file cells-12-02508-s001.zip › Fig. S2.jpg]

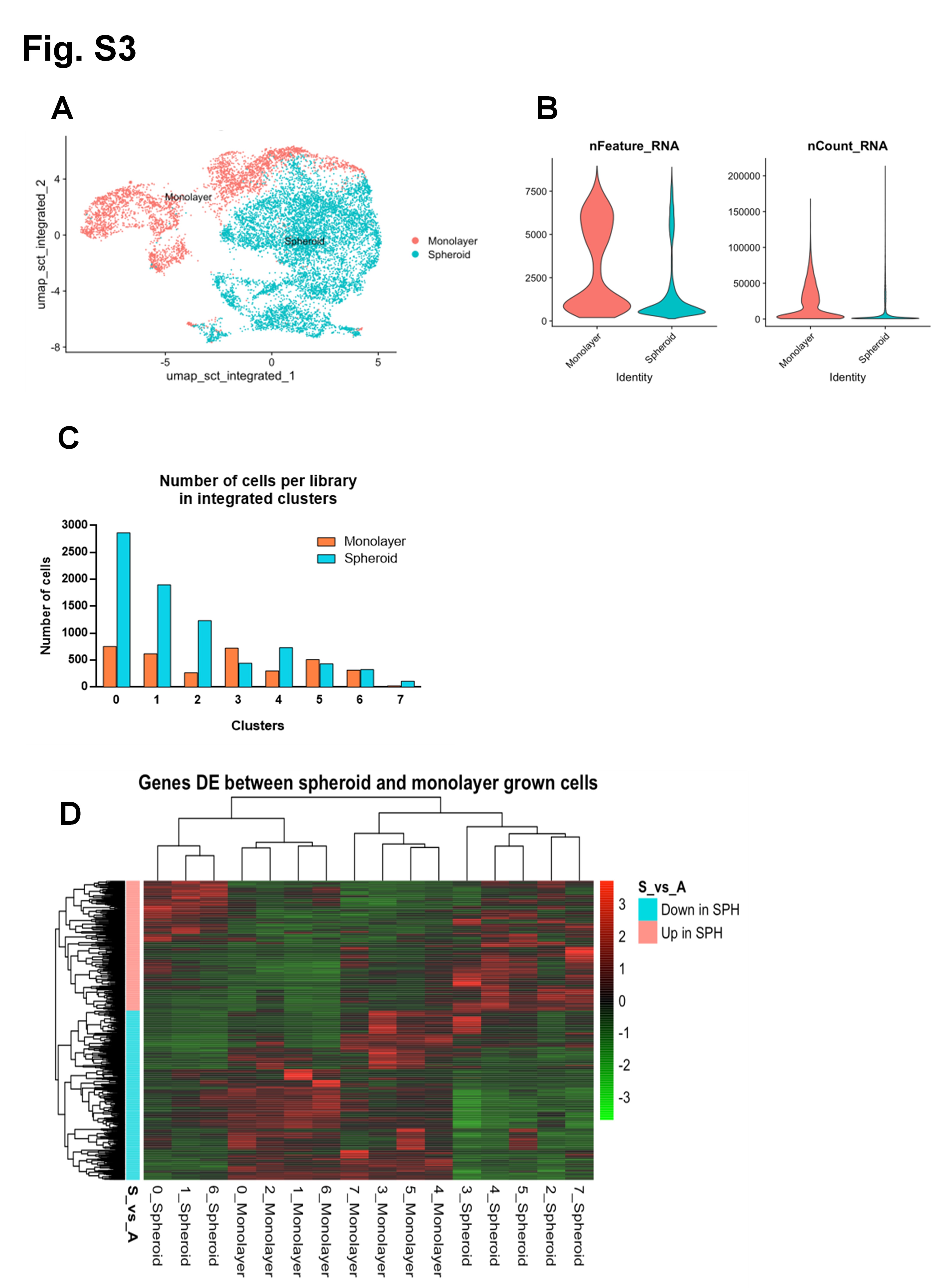

Supplement: Supplementary file 1 [file cells-12-02508-s001.zip › Fig. S3.jpg]

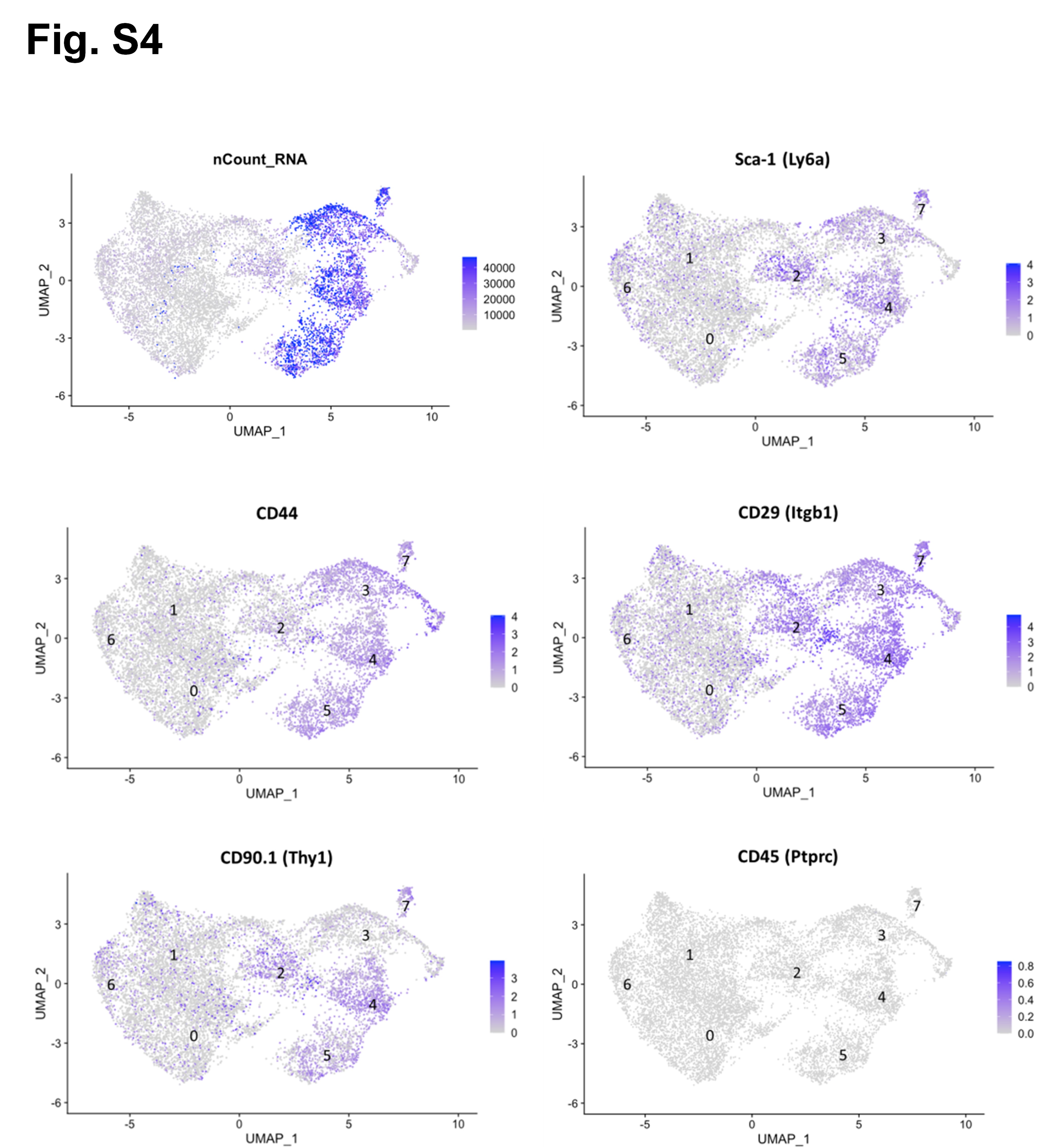

Supplement: Supplementary file 1 [file cells-12-02508-s001.zip › Fig. S4.jpg]

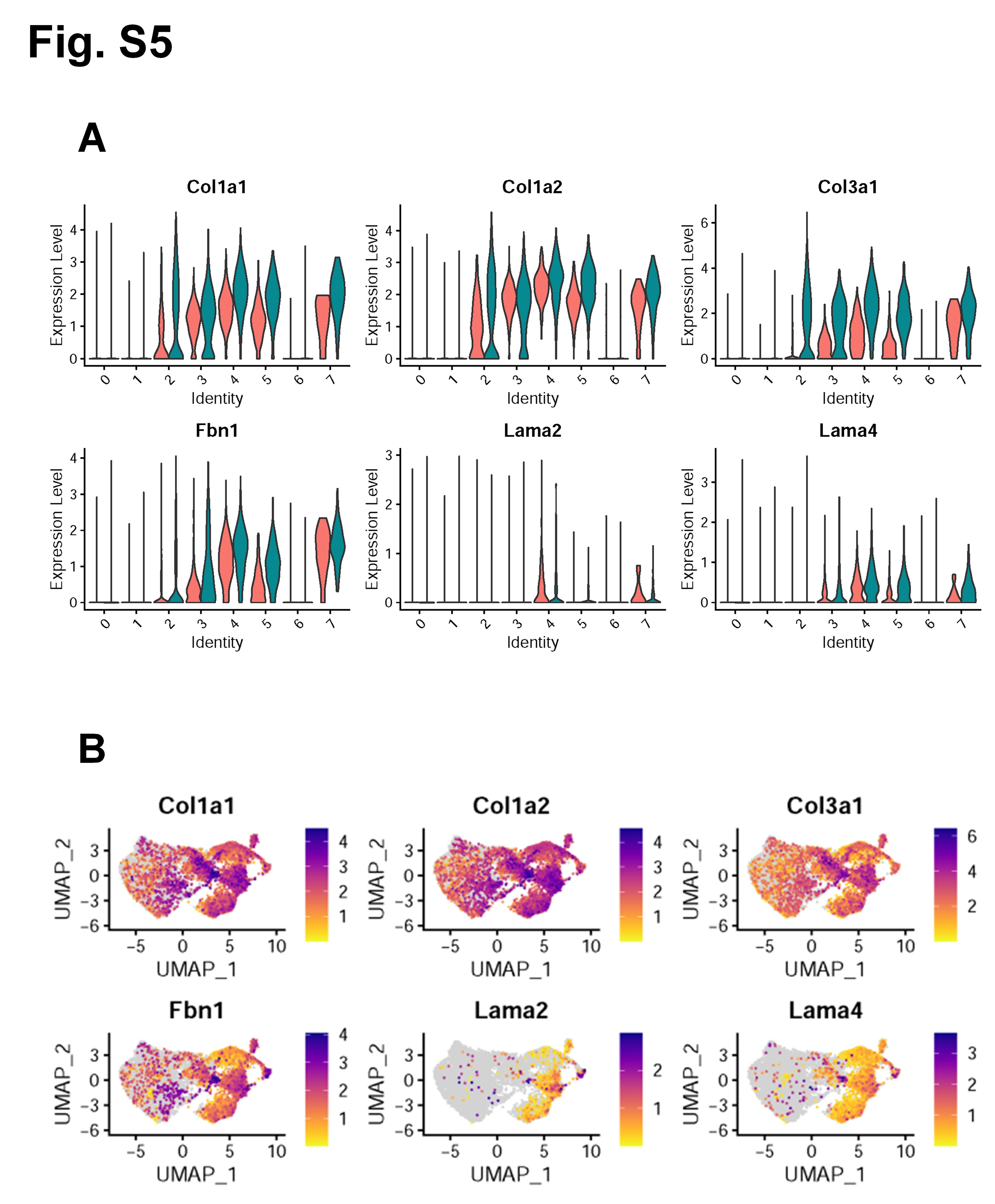

Supplement: Supplementary file 1 [file cells-12-02508-s001.zip › Fig. S5.jpg]

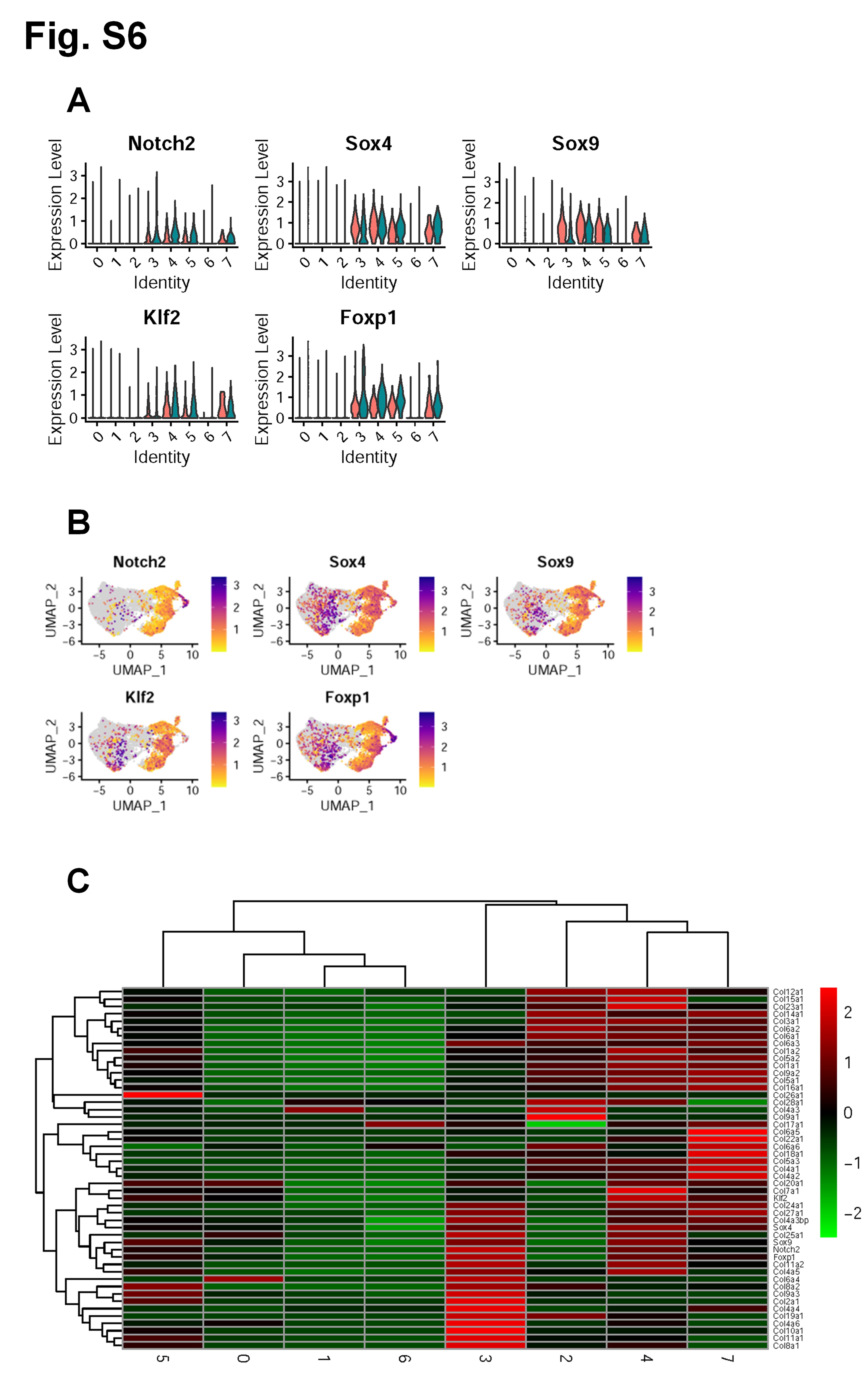

Supplement: Supplementary file 1 [file cells-12-02508-s001.zip › Fig. S6.jpg]

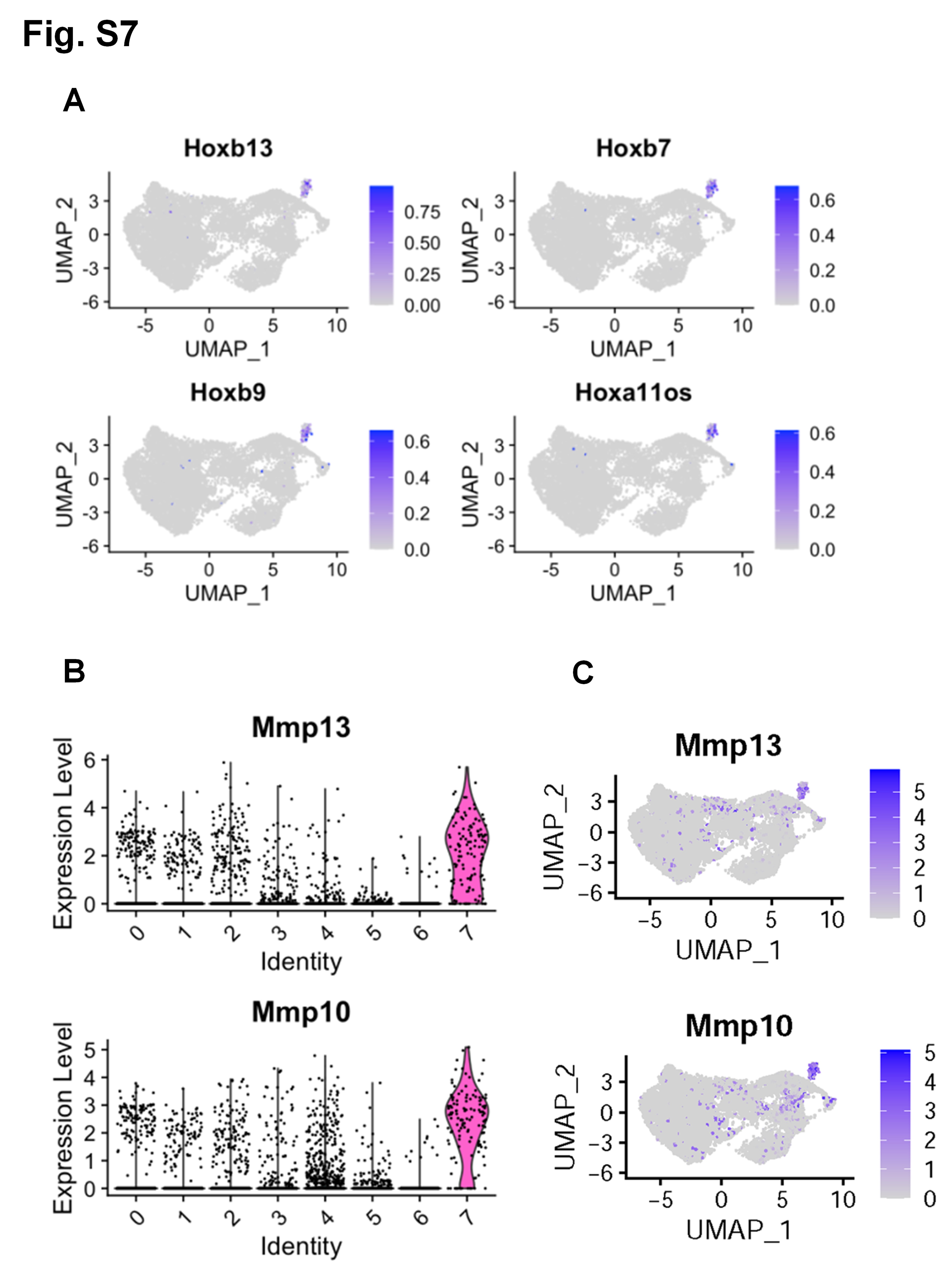

Supplement: Supplementary file 1 [file cells-12-02508-s001.zip › Fig. S7.jpg]

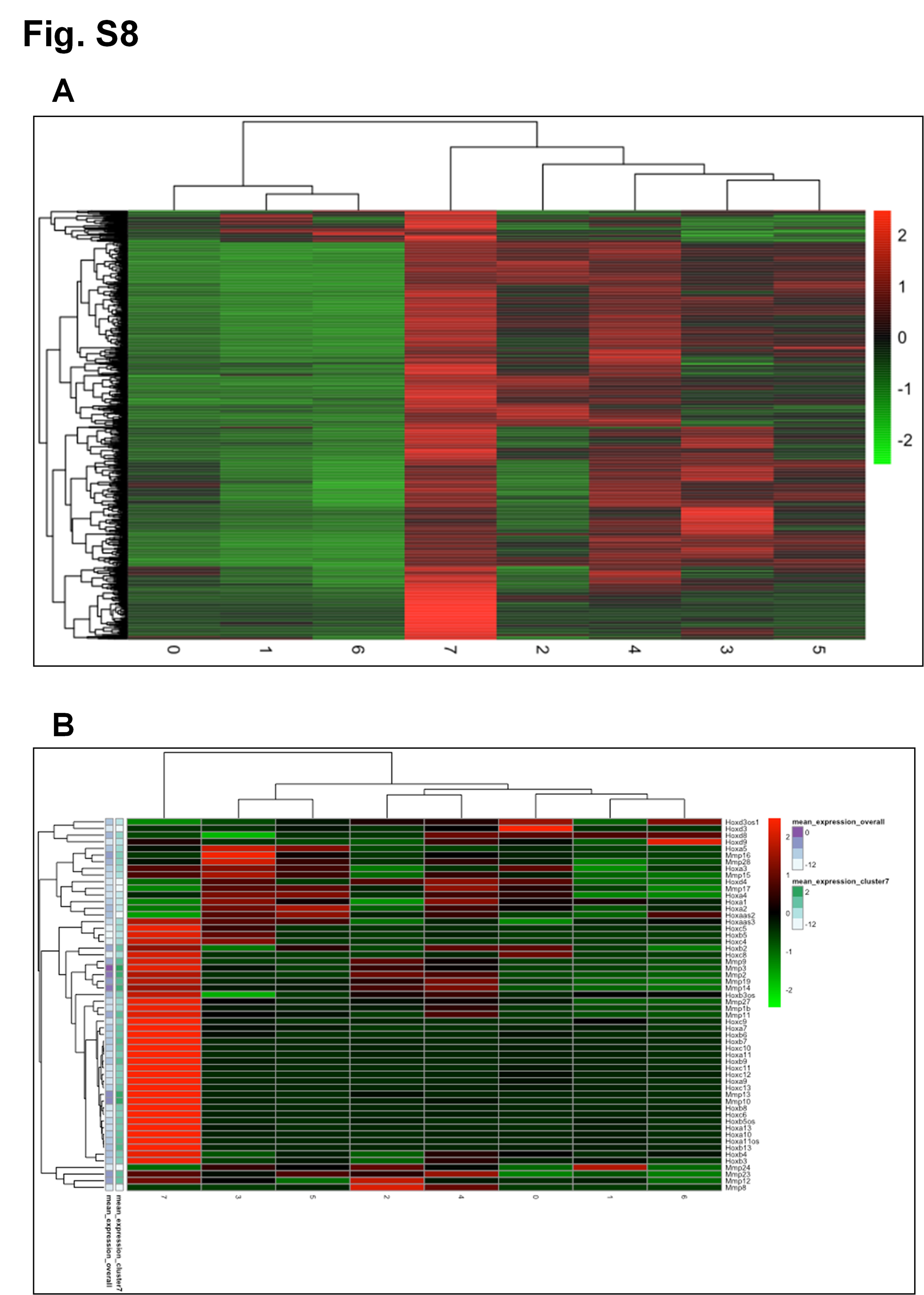

Supplement: Supplementary file 1 [file cells-12-02508-s001.zip › Fig. S8.jpg]

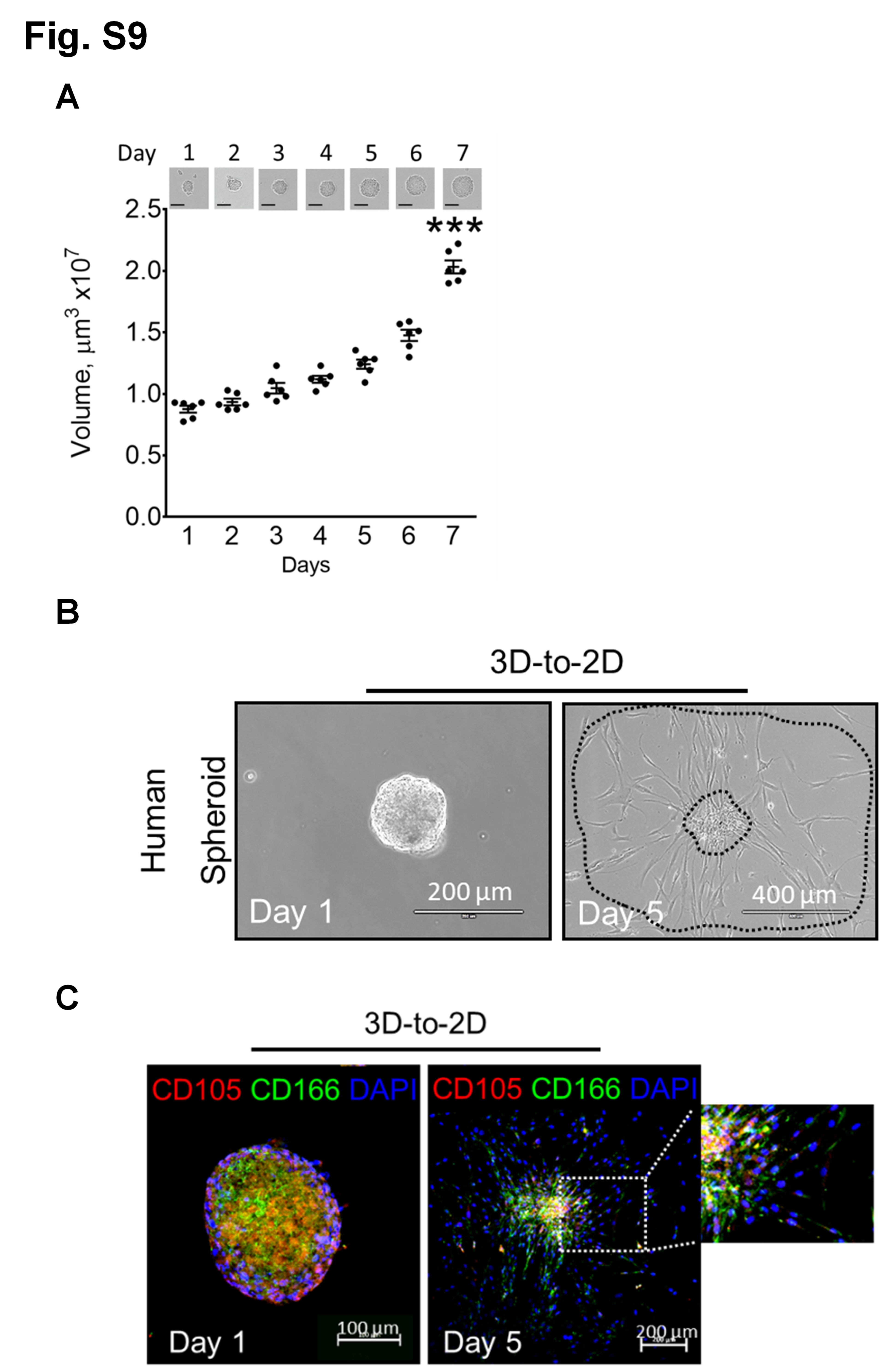

Supplement: Supplementary file 1 [file cells-12-02508-s001.zip › Fig. S9.jpg]
